# A novel model on time-resolved photoluminescence measurements of polar InGaN/GaN multi-quantum-well structures

**DOI:** 10.1038/srep45082

**Published:** 2017-03-22

**Authors:** Yuchen Xing, Lai Wang, Di Yang, Zilan Wang, Zhibiao Hao, Changzheng Sun, Bing Xiong, Yi Luo, Yanjun Han, Jian Wang, Hongtao Li

**Affiliations:** 1 Tsinghua National Laboratory for Information Science and Technology, Department of Electronic Engineering, Tsinghua University, Beijing 100084, China

## Abstract

Based on carrier rate equation, a new model is proposed to explain the non-exponential nature of time-resolved photoluminescence (TRPL) decay curves in the polar InGaN/GaN multi-quantum-well structures. From the study of TRPL curves at different temperatures, it is found that both radiative and non-radiative recombination coefficients vary from low temperature to room temperature. The variation of the coefficients is compatible with the carrier density of states distribution as well as the carrier localization process. These results suggest that there is a novel method to calculate the internal quantum efficiency, which is a complement to the traditional one based on temperature dependent photoluminescence measurement.

Significant progress has been made during the past decades in InGaN/GaN multi-quantum-well (MQW) light emitting diodes (LEDs), which show great potential in applications such as solid state lighting and high brightness display^1^. In order to increase the device performance, it is crucial to understand the carrier recombination mechanism of the materials, and find a reliable method to determine the internal quantum efficiency (IQE). Previously, most IQE calculations are based on temperature-dependent photoluminescence (PL) measurement^2^,^3^,^4^,^5^, the heart of which is the normalization of integrated PL intensity to the data at low temperature (LT). This method assumes that nonradiative recombination is negligible at low temperature. However, as the carriers still have a low and non-zero probability to be captured by the nonradiative recombination centers at LT, the assumption lacks justification. Moreover, it is generally accepted that IQE is a function of the carrier concentration, which is usually unspecified in this method due to the lack of calculating related carrier recombination coefficients. Also there are some other reports on IQE measurement related to the rate equation analysis^6^,^7^,^8^. It should be noted that all the works mentioned above need several PL measurements with varied condition, such as temperature or excitation power.

Another promising method to obtain IQE is TRPL measurement, the result of which can directly reveal the decay of PL emission, leading to a profound understanding of the recombination process of carriers^3^,^9^,^10^,^11^,^12^. The pioneering work in 2000^13^ suggested that the TRPL decay curves of InGaN/GaN quantum wells should be exponential due to exciton recombination. But recently there are a number of publications revealing that in polar and III-N multi-quantum-well (MQW) structures, such as InGaN/GaN and GaN/AlGaN, the TRPL curves are non-exponential at both low temperature^3^,^9^,^14^,^15^ and room temperature^10^,^16^. Proposed by Morel *et al*. is one possible explanation that the non-exponential decay is due to an “pseudo-DAP” recombination^15^. The model uses a phenomenological parameter *η*, whose physical meaning is unclear, to fit the non-exponential nature and only deals with the low temperature condition. Also there are some reports attributing this nature to a bi-exponential decay. The work on ultraviolet MQW carried out by Iwata *et al*.^9^ suggested that the fast and slow decay components were due to nonradiative and radiative recombination respectively, and the non-exponential nature indicates that IQE is quite low instead of 100% at LT. Ngo *et al*. applied the same model to yellow-green MQW structures^3^. However, as the blue MQWs with medium wavelength also exhibit apparent non-exponential decay at low temperature, they should follow the same conclusion, which is contradictory to the general opinion that blue MQW structures usually have very high IQE. Besides, there are some reports using stretched-exponential model^17^ to fit the non-exponential curve. But as there exists a *β* parameter representing the obscure admixture of different exponential components, it is much more like a mathematical fitting method in the absent of explicit discussion of the recombination process. In summary, there is still a controversial debate on the exact mechanism behind the non-exponential nature, and consequently lacks a convincible method to calculate IQE accurately in the TRPL measurement. However, considering that the non-exponential nature does not appear in the non-polar InGaN/GaN structures^14^ but only exists in the polar and semipolar (though not very obvious) ones^16^,^18^,^19^, a great possibility is that it results from the polarization electric fields, which will cause quantum confined stark effect (QCSE). The distribution of electrons and holes is separated, influencing the behavior of their recombination^20^,^21^,^22^.

In this work, a new model based on carrier rate equation is established to explain the non-exponential nature of the TRPL decay curves in polar InGaN/GaN MQW structures. First, the model will be presented theoretically, and then it is applied to analyze TRPL experimental results. Finally, a new method based on it is developed for the IQE calculation.

## Experiment

All the samples discussed in this work are grown on c-plane patterned sapphire substrates by metal-organic chemical vapor deposition (MOCVD). Sample A is a blue InGaN/GaN MQW structure and its growth details have been described elsewhere^23^,^24^. It consists of a 4-μm undoped GaN bulk layer, a 60-nm In_0.02_Ga_0.98_N underlying layer, and 5 pairs of In_0.2_Ga_0.8_N (2.5 nm)/GaN (22 nm) MQW. Samples B and C are commercial blue and green LED wafers with the state-of-the-art efficiency, respectively. Their main epitaxial structures include an n-GaN bulk layer, n-InGaN/GaN superlattices for prestrain, 12 pairs of InGaN (2.5~3 nm)/GaN (10 nm) MQW with the indium composition of about 15~25%, a p-AlGaN electron blocking layer and a p-GaN contact layer. The peak wavelength of samples A, B, and C is 446, 451, and 538 nm, respectively.

A picosecond laser diode (wavelength: 399.6 nm, pulse duration: 70 ps, repetition rate: 80 MHz, mean power: 44 mW) is employed as the excitation source in PL and TRPL measurement. During all the measurements, the repetition rate is reduced to 2.5 MHz by a pulse picker. The luminescence is dispersed by a 55-cm monochromator and detected by a GaAs photocathode photomultiplier tube (PMT) to obtain the spectra. In TRPL measurement, a time-correlated-single-photon-counting (TCSPC) system is used. The PL signals are dispersed by a 32-cm monochromator and temporally resolved by a micro-channel-plate PMT. The measurements are performed at a cryogenic temperature from 5 to 300 K.

## Theoretical Model

In the model established by Chichibu, S. F. *et al*. in 2000^13^, the exciton recombination in InGaN/GaN MQWs should follow the rate equation:


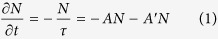


where *N* represents the exciton concentration, *τ* = 1/(*A* + *A*′) represents the carrier lifetime, *A* and *A′* represent radiative and nonradiative recombination coefficient respectively. The solution of equation (1) is


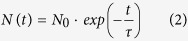


where the decay curve of emitted photons should be exponential.

However, as there are strong intrinsic electric fields in polar InGaN/GaN MQW structures, the electrons and holes could be separated spatially in the direction perpendicular to the plane of QWs, which reduces the correlation between the electron-hole pairs and leads to a recombination nature deviating from the exciton recombination described by the above model^25^,^26^. As a result, the radiative recombination would gradually approximate a bimolecular recombination with the decreasing correlation, and the decay of carrier concentration would no longer follow equation (1).

**Figure 1:**
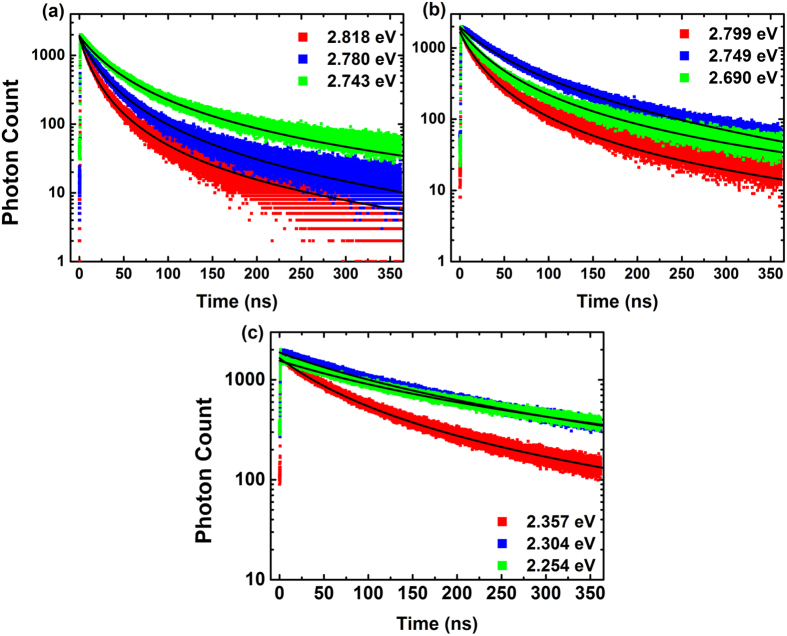
Typical TRPL decay curves of Samples A (**a**), B (**b**) and C (**c**) at low temperature (5 K). Three decay curves with different photon energies are illustrated for each sample: the red, blue and green scatter plot corresponds to higher energy side, the peak energy and lower energy side of the 0-phonon peak, respectively. The black line is the fitting result of each decay curve.

Figure 1 shows non-exponential TRPL decay curves of samples A, B and C at low temperature (5 K). For each sample, three photon energies are chosen for illustration across the 0-phonon peak: one at the peak energy, another at the higher energy side, a third at the lower energy side. Similar results can also be found in many publications.

It is widely accepted that there are mainly three carrier-recombination mechanisms in InGaN/GaN MQW structure: Shockley-Read-Hall (SRH) nonradiative recombination, bimolecular radiative recombination and Auger nonradiative recombination. These mechanisms are usually expressed by *An, Bn*^2^, *Cn*^3^ respectively, where *A, B, C* denote recombination coefficients and *n* denotes carrier concentration. The Auger recombination plays a significant role only at high carrier concentration^27^,^28^,^29^,^30^, hence can be neglected in our photon-excited PL measurements where the carrier concentration is not high enough. Thus in our experiments the rate equation could be expressed as:


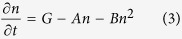


wherein the radiative recombination is described as classic bimolecular recombination *Bn*^2^ in comparison with equation (1). Here *G* represents the carrier generation rate. In TRPL measurements the luminescence is excited by ~ps laser pulse, so there is *G* = 0. And we could solve equation (3):


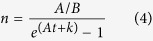


where *k* is a constant decided by the initial value of *n*. As the number of emitted photons *i* is only related to radiative recombination, *i.e. i* ∝ *n*^2^.

We get


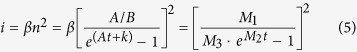


where *M*_*1*_, *M*_*2*_, *M*_*3*_ are fitting parameters. Equation (5) is the expression of TRPL decay curves. From the fitting parameters and initial relationship 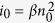 (*i*_*0*_ represents the number of emitted photons at *t* = 0 and *β* is a proportional coefficient) we can get the following coefficients


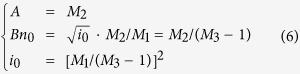


where *A* represents the nonradiative recombination coefficient, while *Bn*_*0*_ represents the initial radiative recombination rate at *t* = 0. Substituting *M*_*1*_, *M*_*2*_, *M*_*3*_ in Equation (5) with Equation (6) the fitting parameters can be transmitted into *A, Bn*_*0*_ and *i*_*0*_. And the expression of decay curves can also be written as


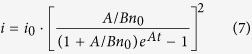


It is clear that the time variable, *t*, only exists in the term (1 + *A*/*Bn*_0_)*e*^*At*^, which implies that the shape of decay curve is decided by *A* and *Bn*_*0*_. *i*_*0*_ is only relevant to the relative amplitude of the decay curves and decided by the integration time of TCSPC system.

It should be noted that in our TCSPC system, only the decay of photons with a certain energy could be measured each time. The rate equations above have neglected the transition between carriers with different energies. However, as long as the energy of the photons is not much higher than the peak of spectrum, the related carriers locate near the minima of the energy band where the empty ‘tail states’ below it is limited, and the recombination process is much stronger than the transition process. Moreover, if the energy of the photons is chosen much lower than the peak, the carriers will come from not only 0-phonon peak but also 1st LO-phonon peak, so the decay curve contains the nature of two carrier groups and cannot be described by one rate equation. Thus, it is reasonable to apply the equation (7) to the emitted photons whose energies are around the PL peak. As a matter of fact, good fitting results of TRPL curves are obtained as shown in Fig. 1.

Also note that in multi-exponential model, each exponential component needs two fitting parameters, *i.e.* amplitude and lifetime. Although there are only *A* and *Bn*_*0*_ involved in our model, excellent fitting results are obtained for all TRPL decay curves, as shown in the next section.

## Results and Discussion

**Figure 2:**
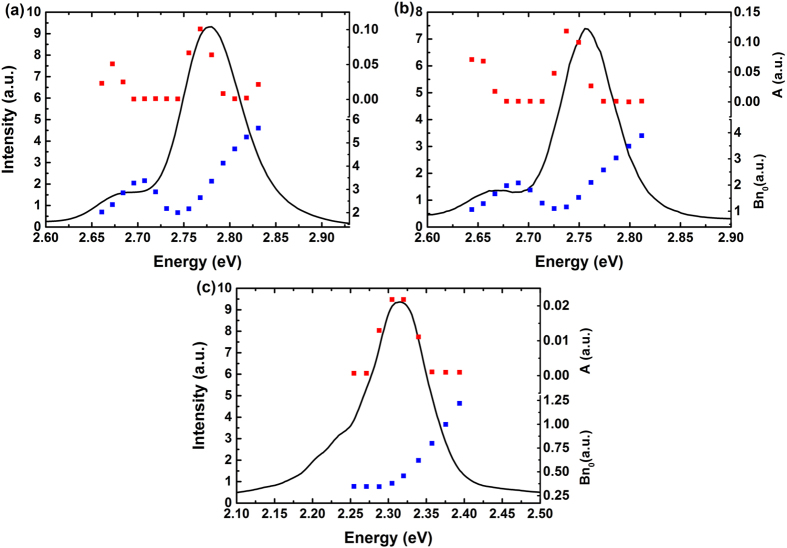
The PL spectrum and model fitting results of Sample A (**a**), B (**b**) and C (**c**) at 5 K. The red and blue dots denote *A* and *Bn*_*0*_, respectively. Note that the values of *A* and *Bn*_*0*_ are comparable in each subfigure though both use arbitrary units.

In Fig. 2 is shown the spectra and fitting results of two blue-emitting (Samples A and B) and one green-emitting (Sample C) samples at 5 K. It is generally accepted that the main peak is assigned to radiative recombination of localized carriers, while the series located at lower energies with a periodicity of ~90 meV are due to longitudinal optical (LO) phonon replicas^31^. The fitting results of *A* and *Bn*_*0*_ also show the same periodicity.

*A* represents the nonradiative recombination coefficient. For most energies, *A* is close to 0 but rises up a little at the lower energy side of the PL peak at 5 K. This may be related to the energy distribution of the SRH recombination centers, by which carriers with certain energies are captured more easily. Still, the exact mechanism needs further investigation.

As mentioned in the previous section, the invalidity of exponential equation implies that the radiative recombination rate is no longer proportional to the carrier concentration but proportional to the square of carrier concentration (*i.e. Bn*^2^ in Equation (3)). From equation (3) it can be seen that the nonradiative recombination coefficient has a dimension of s^−1^. For the convenience of comparison between the nonradiative recombination and the radiative one, a quantity of the same dimension is necessary. As the dimension of *Bn* is also s^−1^, we define its initial value, *Bn*_*0*_, as an indicator for the relative change of radiative recombination among different TRPL curves. The comparison between *A* and *Bn*_*0*_ can reflect the relationship between the two recombination mechanisms. As photon energy increases, *Bn*_*0*_ rises up across the main emission peak in all samples as shown in Fig. 2. The trend is consistent with the picture of QCSE in the MQW structures: The overlapping of the wavefuntions of electrons and holes increases with their energy, giving more chances for radiative recombination, *i.e.* increasing the coefficient. From the comparison between the subfigures in Fig. 2, it is obvious that the *Bn*_*0*_ is higher in the blue-emitting samples A and B than the green-emitting sample C. It is compatible with the fact that in the green-emitting samples the radiative recombination rate is lower than that in the blue-emitting ones because of stronger QCSE. Assuming that *B* is constant, a larger *Bn*_*0*_ can be expected under a higher laser pulse energy which provides larger *n*_*0*_. As a result, a more rapid decay should present at the beginning of the TRPL curve. Although the excitation power density cannot vary in our experiment, the prediction is compatible with previous study (see Fig. 3 in ref. 32).

Due to the low temperature at 5 K, the SRH recombination centers are scarcely activated, leading to a nonradiative recombination coefficient which is much lower than the radiative counterpart. When the temperature increases, both will change dramatically as shown below, where Sample A is used as an example.

**Figure 3:**
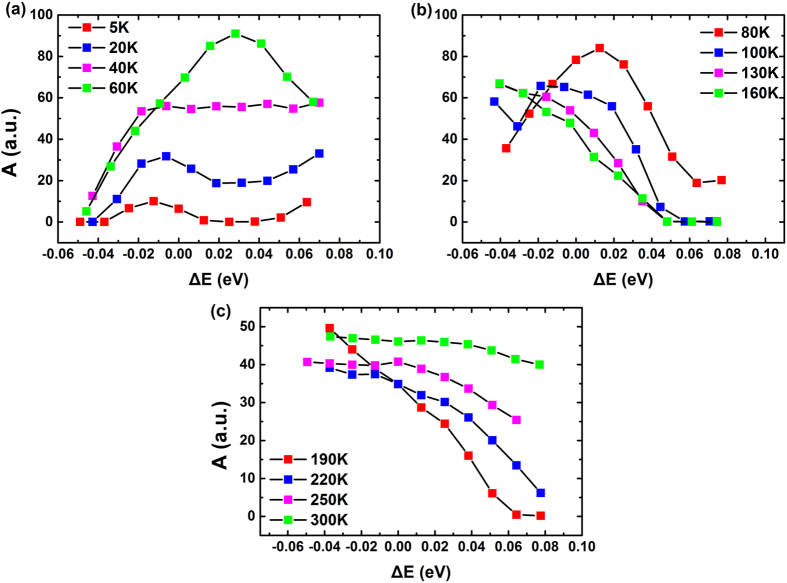
The temperature dependence of nonradiative recombination coefficient *A* from 5 K to 300 K for sample A. (**a**) 5–60 K (**b**) 80–160 K (**c**) 190 K–300 K. Considering the ‘S-shaped’ shift of emission peak relative to temperatures, the horizontal axis *ΔE* denotes the energy difference between the photons detected and the PL main peak, and *ΔE* = 0 represents the PL peak energy of each temperature.

Figure 3 exhibits the temperature dependence of *A* for Sample A. Here, Δ*E* = *E*_*photon*_ − *E*_*peak*_ is calculated to define the energy difference between the detected photons (*E*_*phonon*_) and the PL main peak (*E*_*peak*_). As the PL peak energy shifts with temperature, the data among different temperatures are aligned according to the PL peak energy, *i.e. ΔE* = 0, for a clear comparison. The detailed *E*_*peak*_ at different temperatures can be found in supplementary information. In the InGaN/GaN MQWs, it is widely accepted that the carriers are localized due to the fluctuation of alloy composition or well width^14^. There is a certain energy distribution of the localization centers, of which the degree of localization differs from each other^33^. For the high-degree localized carriers (*e.g*. with deeper localization depth), the nonradiative recombination probability is lower than those low-degree localized^34^, mainly because the nonradiative recombination in the localization centers is more suppressed^5^,^35^,^36^,^37^,^38^. At low temperature, all carriers are nearly “frozen” at where they are generated and cannot transfer to other places easily. When thermalized, there are two factors which can influence the behavior of carrier recombination. First, more and more carriers are able to move and prefer to distribute in the high-degree localization centers. Second, the nonradiative recombination centers are gradually activated at the same time. At 0–60 K, the second factor plays a more important role, because the transfer of carriers is not obvious due to their immobility at low temperature, while the probability of nonradiative recombination increases with the temperature regardless of the localization degree. As a result, the overall coefficient *A* increases. However, when the temperature increases to 60–190 K, the impact of the transfer between localization centers dominates and more carriers can move to the high-degree localization centers, where the probability of nonradiative recombination is much lower. Thus, the overall coefficient *A* decreases. As the temperature exceeds 190 K, there is a delocalization process induced by the far more thermalized carriers, and they gradually escape from localization centers, which results in a higher chance of being captured by nonradiative recombination centers. And the value of *A* increases again consequently.

**Figure 4:**
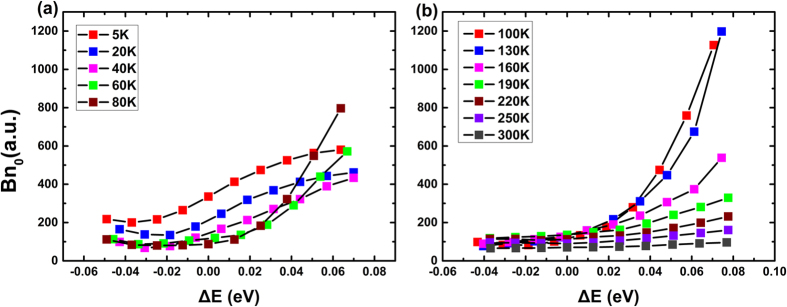
The temperature dependence of initial radiative recombination coefficient *Bn*_*0*_ from 5 K to 300 K for sample A. (**a**) 5–80 K (**b**) 100–300 K. Considering the ‘S-shaped’ shift of emission peak relative to temperatures, the horizontal axis *ΔE* denotes the energy difference between the photons detected and the PL main peak, and *ΔE* = 0 represents the PL peak energy of each temperature.

The temperature dependence of *Bn*_*0*_ for Sample A is shown in Fig. 4. For the convenience of illustration, the variation is depicted in two subfigures. Although rises slightly from 80 K to 130 K at around *ΔE* = 0, it is obvious that *Bn*_*0*_, the indicator of radiative recombination coefficient, keeps decreasing with the increasing temperature. It should be noted that although *Bn*_*0*_ increases from 40 to 100 K at high energy side of the main PL peak, it contributes little to the entire behavior of emitted photons because of the low PL intensity at this energy range.

The main decreasing trend of *Bn*_*0*_ is due to the energy distribution of the density of states (DOS) of carriers in quantum well structure, which is expressed by *D*_2*D*_(*E*) ∝ *const*. According to the study of J. Feldmann *et al*.^39^, the thermalization reduces the fraction of excitons within the spectral width that can contribute to the radiative recombination, which results in an increasing decay time, *i.e.* a decreasing recombination coefficient. Although the separate distribution of the electrons and holes in polar InGaN/GaN MQW structures will change the nature of recombination, that main trend won’t be affected. As a matter of fact, the experimental results of Chichibu *et al*. also confirmed the slowing down of the radiative recombination with the increasing temperature^13^.

## IQE calculation

The final task of our model is to calculate the IQE of InGaN/GaN MQW structure. With the rate equation (Equation (3)) and its solution (Equation (4)) obtained previously, the carriers can be clearly divided into nonradiative and radiative recombination. The respective integration on time are as follows:


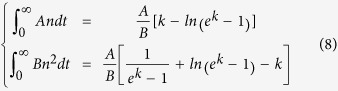


Considering


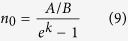


the IQE can be expressed as


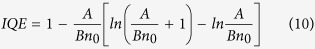


The efficiency is decided by three parameters: nonradiative recombination coefficient *A*, radiative recombination coefficient *B*, as well as initial carrier concentration *n*_*0*_. Note that *n*_*0*_ is the carrier concentration injected by each laser pulse in TRPL measurement, suggesting that the IQE will increase with higher excitation power.

**Figure 5:**
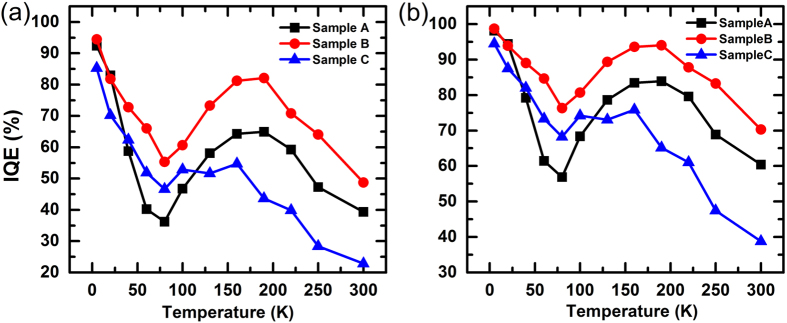
The temperature-relative IQE of Sample A, B and C, calculated from the TRPL decay curves at the energy of PL main peak. The detailed decay curves and fitting results can be found in the supplementary information. (**a**) The IQE calculated by Equation (10), (**b**) the IQE calculated by *Bn*_*0*_/(*A* + *Bn*_*0*_) which simulates an IQE under a constant carrier concentration *n*_*0*_.

As mentioned above, only the decay curve of one wavelength is measured each time in TCSPC system. Some reports^16^,^18^ use the data of peak wavelength to indicate the property of whole spectra in TRPL measurements. Applying Equation (10) to the peak-wavelength data of blue-emitting Sample A & B and green-emitting Sample C, the respective calculated IQE for LT/RT are 92.4%/39.3%, 94.4%/48.7% and 85.4%/22.8%. The IQE of each temperature is depicted in Fig. 5(a). The detailed information of the TRPL decay curves can be found in supplementary information. Simulating the steady excitation condition where (1) the carrier concentration is constant at *n*_*0*_ and (2) the respective recombination coefficient is the same as *A* and *B* in Fig. 5(a), a general calculation method *IQE* = *Bn*_0_/(*A* + *Bn*_0_) is utilized and the results are shown in Fig. 5(b). The respective IQE for LT/RT are 98.1%/60.4%, 98.8%/70.3% and 94.4%/38.8% for sample A, B and C.

The calculation through *IQE* = *Bn*_0_/(*A* + *Bn*_0_) shows that the IQE at low temperature (5 K) can be nearly 100% for samples A and B as expected. However, it is not the case for sample C (94.4%), which implies the IQE calculation through normalization of PL intensity at LT is not exactly accurate in temperature dependent PL measurement. From Fig. 2 we can see that both parameters *A* and *Bn*_*0*_ in sample A and B are much larger than those in sample C, and it is their relative values that determine the IQE. Also, the IQE of the three samples do not decrease monotonically with increasing temperature as what is expected in temperature dependent PL measurement. The discrepancy could result from the difference of the excitation condition. In Fig. 5(a), the IQE is calculated under transient excitation with a certain pulse energy. In TRPL measurement, the carrier concentration keeps decreasing after the transient excitation, and the radiative recombination rate *Bn*^2^ decreases faster than the nonradiative rate *An*. While in the steady-excited temperature dependent PL measurement, both recombination rates keep constant. As a result, the fraction of radiative recombination in TRPL measurement is respectively lower, leading to a lower IQE in Fig. 5(a) than Fig. 5(b). From the previous section, we can attribute the increasing IQE at 80–190 K to the relative variation of *A* and *Bn*_*0*_. Though the radiative recombination gradually becomes slower, the rate of nonradiative recombination decreases more rapidly. As a consequence, more and more carriers recombine radiatively as temperature increases. Although in Fig. 5(b) a steady excitation is assumed, there are still differences from traditional temperature dependent PL measurement: The IQE calculated at different temperatures actually corresponds to a constant carrier concentration *n*_*0*_, while its counterpart in temperature dependent PL measurement corresponds to a constant carrier generation rate *G*.

Finally, it should be noted again that IQE is closely related to the excitation condition. Discussion on IQE in absence of carrier concentration usually means little, since IQE can always be increased through higher carrier concentration as long as efficiency droop effect doesn’t occur, even the sample is of poor quality. Therefore, although the value of IQE is an important criterion to qualify the InGaN/GaN MQWs, what matters most is to figure out the relationship between radiative and nonradiative coefficients, which can give a more profound understanding to the carrier recombination mechanism and provide a clear comparison between the quality of different samples. As our model is based on TRPL measurement, the calculated IQE corresponds to the pulsed laser excitation. Moreover, the *Bn*^2^ approximation in our model is valid for radiative recombination in polar InGaN/GaN MQW structures because of the small Coulomb interaction energy caused by QCSE. When it goes to nonpolar or semipolar structures, or samples with low indium composition where QCSE is quite small, the classic excitonic recombination described by Equation (1) still needs to be considered.

## Summary

In conclusion, we have proposed a novel theoretical model to explain the non-exponential decay curve of TRPL measurements for polar InGaN/GaN MQW structures. The model is based on carrier rate equation which includes SRH nonradiative recombination and bimolecular radiative recombination. Applied on TRPL data of polar blue and green InGaN/GaN MQW, the model obtains excellent fitting results for nonradiative recombination coefficient *A* and initial radiative recombination parameter *Bn*_*0*_. From the study of the temperature dependence of *A* and *Bn*_*0*_, it is suggested that the thermal activation of SRH recombination centers is influenced by carrier localization and delocalization process, which will cause a decreasing nonradiative recombination rate from 60 to 190 K. Meanwhile, the radiative recombination gradually slows down with increasing temperature, mainly due to the energy distribution of carrier DOS in quantum wells. The application of the model to IQE calculation is also carried out on polar InGaN/GaN MQWs. Instead of decreasing monotonically, IQE will rise in the mid-range of the temperature, mainly attributed to the rapid decrease of nonradiative recombination rate. Also, it is found that the assumption that there is no nonradiative recombination at low temperatures may not be fully valid. As our model studies pulsed excitation condition where the carrier concentration decays fast, it could shed light on the carrier recombination dynamics of high speed modulated InGaN/GaN MQW LEDs. The calculation method based on coefficients *A* and *B* derived directly from the TRPL curve also provides a new prospect for RT IQE measurement.

## Additional Information

**How to cite this article:** Xing, Y. *et al*. A novel model on time-resolved photoluminescence measurements of polar InGaN/GaN multi-quantum-well structures. *Sci. Rep.* **7**, 45082; doi: 10.1038/srep45082 (2017).

**Publisher's note:** Springer Nature remains neutral with regard to jurisdictional claims in published maps and institutional affiliations.

## Supplementary Material

Supplementary Information
