# Supplementary material for: A novel model on time-resolved photoluminescence measurements of polar InGaN/GaN multi-quantum-well structures
Source: Sci Rep. 2017 Mar 22;7:45082. doi: 10.1038/srep45082 (PMC5361181; doi:10.1038/srep45082)
Supplement: Supplementary Information [file srep45082-s1.pdf]

A novel model on time-resolved photoluminescence measurements of polar InGaN/GaN multi-quantum-well structures

Authors: Yuchen Xing[1], Lai Wang[1]*, Di Yang[1], Zilan Wang[1], Zhibiao Hao[1], Changzheng Sun[1], Bing Xiong[1], Yi Luo[1]*, Yanjun Han[1], Jian Wang[1], Hongtao Li[1]

Affiliations:

[1]Tsinghua National Laboratory for Information Science and Technology, Department of Electronic Engineering, Tsinghua University, Beijing 100084, China.

Correspondence and requests for materials should be addressed to L.W. (email: wanglai@mail.tsinghua.edu.cn) or Y.L. (email: luoy@mail.tsinghua.edu.cn)

Supplementary Information

This supplementary Information contains two parts: the first part illustrates the information about temperature dependent PL spectra of sample A; the second part illustrates the detailed decay curves used in Figure 5 of the manuscript entitled “A novel model on time-resolved photoluminescence measurements of polar InGaN/GaN multi-quantum-well structures” (SREP-16-43591).

For a better understanding of Figure 3-4 in the manuscript, Figure S1 shows the PL spectra of sample A at different temperatures. For a better illustration of the peak energy, the max intensity of all spectra are normalized to 100 in Figure S1(a)-(c). Figure S1(d) shows the shifts of PL peak energy E_{peak} against temperature. Instead of acquired through multi-peak fitting method, E_{peak} is defined as the energy position where the PL intensity is maximum, and acquired directly from the experiment data.

Figure S2 and S3 shows the decay curves of the peak energy at 5-300 K for blue-emitting sample A and B, respectively. (For the convenience of illustration, all the TRPL decay curves have been normalized to 100.) Note that for both samples the decay of the TRPL curves is slowing down as the temperature increases. This result is different from the work carried out by Cho *et al.* in 1998[1], where the decay of the photoluminescence (PL) became faster at mid-range temperature. The difference could be generated from the quality of InGaN/GaN samples: If there are lots of SRH centers activated during thermalization, the localization process may not be able to suppress the nonradiative recombination significantly. Thus, the decay may become faster as a result of the increase of *A*. Shown in Table S1 and S2 are the respective fitting parameters *A* and Bn_0 as well as the calculated IQE in Fig.5 of the manuscript for sample A and B. "IQE in Fig 5(a)" is calculated from Equation (10), and “IQE in Fig 5(b)” is calculated from $Bn_0/(A+Bn_0)$.

Figure S4 shows the decay curves of the peak energy at 5-300 K for sample C. Although the decay of the TRPL curves also slows down from 5 K to 190 K, there is a significant decrease of the decay time at 190-220 K. From the fitting results shown in Table S3, this can be attributed to the increase of *A*, which may result from the thermal activation of some kind of nonradiative recombination center in the green-emitting samples. Note that as Bn_0 keeps decreasing, even smaller than *A* at 250-300 K, the bimolecular recombination gradually loses its domination and the non-exponential nature of the decay curves dims.

Reference

1. Cho, Y.-H. et al. ‘S-shaped’ temperature-dependent emission shift and carrier dynamics in InGaN/GaN multiple quantum wells. Applied Physics Letters 73, 1370–1372 (1998).

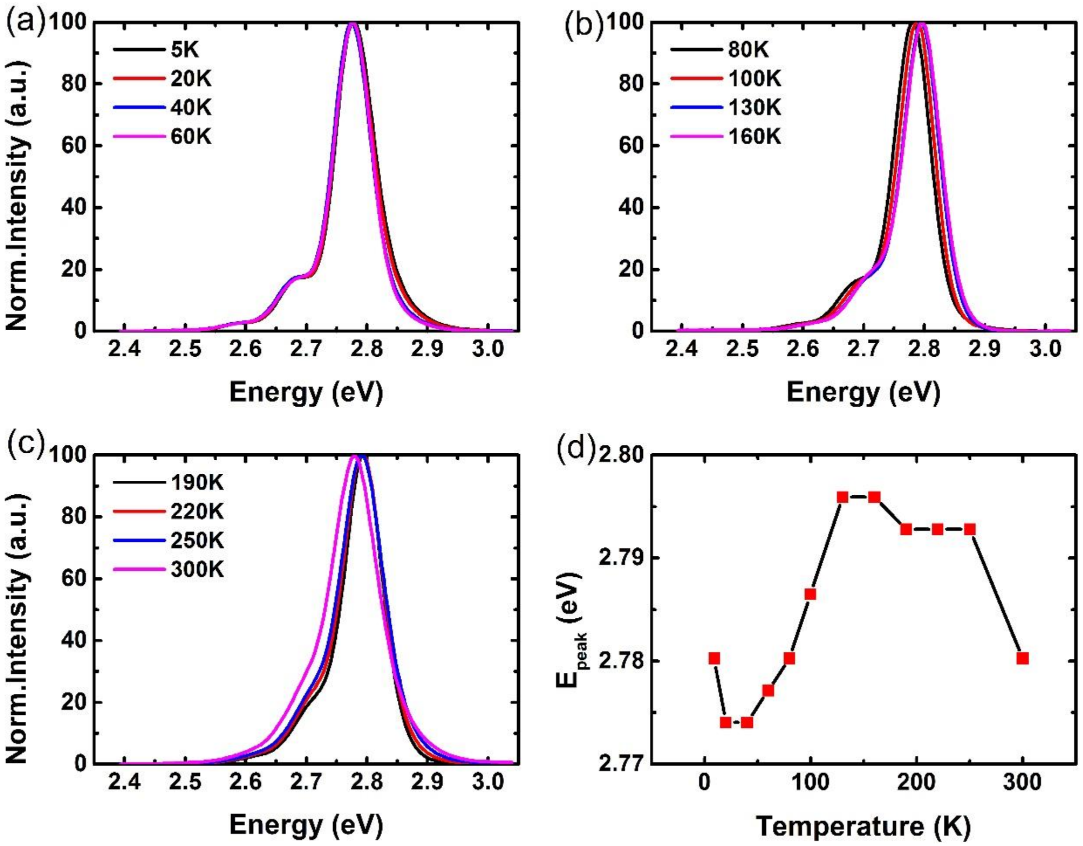

Figure S1. Normalized PL spectra of sample A at different temperatures, where (a) 5-60 K (b) 80-160 K (c) 190-300 K. (d) shows the detailed variation of E_{peak} against temperature.

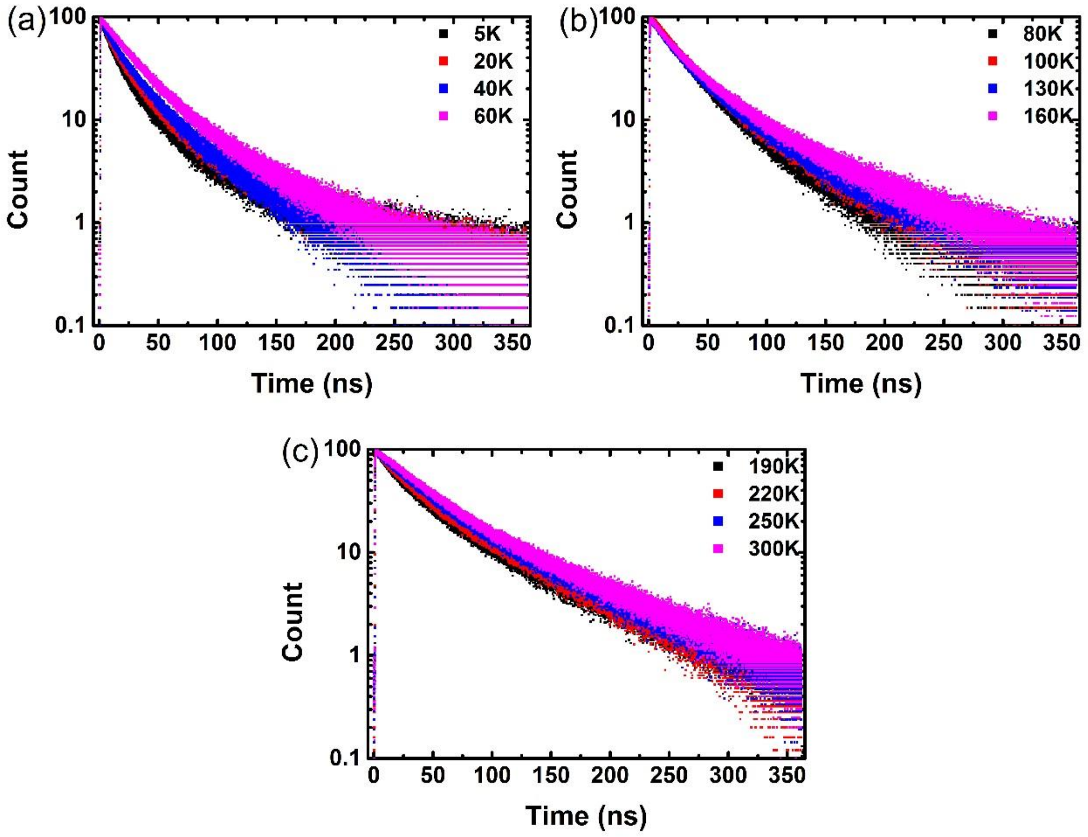

Figure S2. TRPL decay curves of sample A at the energy of PL main peak. (a) 5-60 K (b) 80-160 K (c) 190-300 K. All the curves have been normalized to photon count 100.

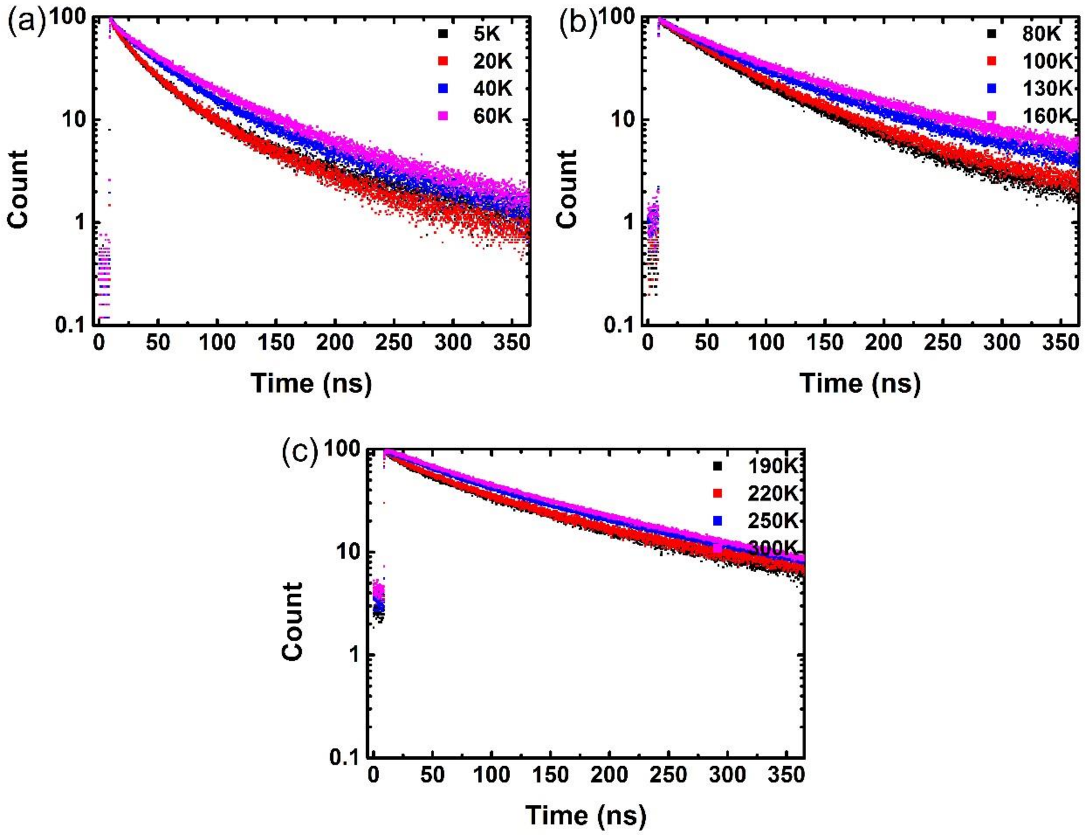

Figure S3. TRPL decay curves of sample B at the energy of PL main peak. (a) 5-60 K (b) 80-160 K (c) 190-300 K. All the curves have been normalized to photon count 100.

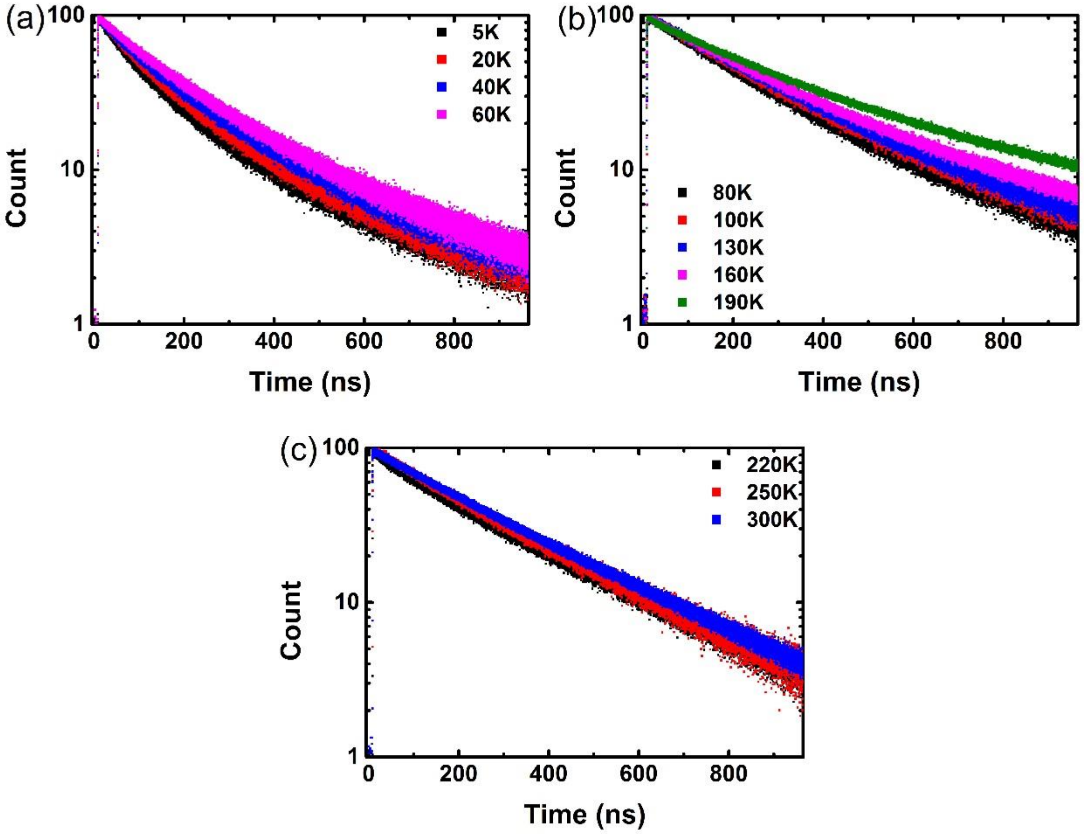

Figure S4. TRPL decay curves of sample C at the energy of PL main peak. (a) 5-60 K (b) 80-190 K (c) 220-300 K. All the curves have been normalized to photon count 100.

Table S1. The fitting parameters A, Bn_0 and calculated IQE (from Equation (10) as well as $Bn_0/(A+Bn_0)$ in the manuscript) of sample A at different temperatures. The TRPL decay curves are from the data acquired at PL main peak.

Temperature (K)	*A* (a.u.)	Bn_0 (a.u.)	IQE in Fig 5(a)	IQE in Fig 5(b)
5	6.38	335.36	92.43%	98.13%
20	18.77	318.14	82.96%	94.43%
40	55.89	212.91	58.77%	79.21%
60	85.03	135.50	40.19%	61.44%
80	84.03	110.94	36.25%	56.90%
100	61.45	132.97	46.77%	68.39%
130	43.03	158.58	58.09%	78.66%
160	31.43	158.24	64.30%	83.43%
190	28.71	149.42	64.93%	83.88%
220	31.94	124.79	59.29%	79.62%
250	40.77	90.30	47.28%	68.89%
300	46.08	70.23	39.25%	60.38%

Table S2. The fitting parameters A, Bn_0 and calculated IQE (from Equation (10) as well as $Bn_0/(A+Bn_0)$ in the manuscript) of sample B at different temperatures. The TRPL decay curves are from the data acquired at PL main peak.

Temperature (K)	*A* (a.u.)	Bn_0 (a.u.)	IQE in Fig 5(a)	IQE in Fig 5(b)
5	2.67	210.00	94.44%	98.75%
20	13.10	200.67	81.77%	93.87%
40	16.36	132.74	72.77%	89.03%
60	18.35	101.28	66.03%	84.66%
80	26.67	85.96	55.30%	76.32%
100	19.18	80.13	60.64%	80.69%
130	9.12	76.34	73.27%	89.33%
160	4.72	69.16	81.24%	93.61%
190	4.26	67.00	82.09%	94.02%
220	7.75	55.94	70.81%	87.83%
250	9.26	46.04	64.07%	83.26%
300	14.48	34.29	48.73%	70.31%

Table S3. The fitting parameters A, Bn_0 and calculated IQE (from Equation (10) as well as $Bn_0/(A+Bn_0)$ in the manuscript) of sample C at different temperatures. The TRPL decay curves are from the data acquired at PL main peak.

Temperature (K)	A (a.u.)	Bn_0 (a.u.)	IQE in Fig 5(a)	IQE in Fig 5(b)
5	2.18	45.80	85.29%	94.46%
20	5.49	38.39	70.28%	87.49%
40	6.63	30.30	62.43%	82.05%
60	8.46	23.20	51.89%	73.28%
80	7.93	17.04	46.62%	68.24%
100	6.06	17.43	52.87%	74.20%
130	6.05	16.41	51.63%	73.06%
160	5.07	15.93	54.77%	75.86%
190	7.94	14.86	43.64%	65.18%
220	9.24	14.47	39.83%	61.03%
250	11.62	10.50	28.38%	47.47%
300	11.86	7.52	22.87%	38.80%
